# ASO Author Reflections: Towards Consensus on Resectability Assessments and Local Treatment Planning for Patients with Initially Unresectable Colorectal Cancer Liver Metastases

**DOI:** 10.1245/s10434-023-13513-4

**Published:** 2023-04-21

**Authors:** Marinde J. G. Bond, Cornelis J. A. Punt, Rutger-Jan Swijnenburg

**Affiliations:** 1 Department of Epidemiology, Julius Centre for Health Sciences and Primary Care, University Medical Centre Utrecht, Utrecht University, Utrecht, The Netherlands; 2 Department of Surgery, Amsterdam UMC, University of Amsterdam and Vrije Universiteit Amsterdam, Amsterdam, The Netherlands; 3 Cancer Center Amsterdam, Amsterdam, The Netherlands

## Past

Local treatment (e.g. surgery and ablation) is the only potentially curative treatment for patients with colorectal cancer liver metastases (CRLM). However, not all patients who may be eligible for local treatment are offered this option. This is due to the absence of transparent and validated criteria for local treatment as well as the inability to keep up with local treatment possibilities by improving surgical techniques such as ablation and liver augmentation in non-liver expert centres. Previous studies have shown that the use of online liver expert panels may increase the number of patients who may benefit from local treatment.^1,2^ However, disagreements in resectability assessments as well as in the type of local treatment have been demonstrated even among experienced liver surgeons.^3–5^ To improve the quality of care for patients with CRLM, more data are required to establish the optimal method of evaluation of these patients and to identify the main factors that contribute to the aforementioned disagreements.

## Present

This study assessed the variability in resectability assessments and local treatment planning among 17 surgeons participating in the Dutch Colorectal Cancer Group (DCCG) liver expert panel from the randomised controlled phase III CAIRO5 study (NCT02162563).^6^ In this RCT, patients with initially unresectable colorectal cancer liver-only metastases were randomised between the currently most effective induction regimens consisting of chemo- and targeted therapy. The analysis of 1149 evaluations of 496 patients showed disagreement between panel surgeons in 50% of resectability assessments, and surgeons proposing different local treatment plans in 77% of patients. The most pronounced inter-surgeon differences concerned the advice to proceed with a major hepatectomy versus parenchymal-preserving approaches involving local resection and ablation strategies.

## Future

Several steps should be taken to reduce this unwarranted variability and to ensure that every patient who is eligible for local treatment is given the opportunity to undergo such treatment. First, all patients with CRLM should be evaluated by a multidisciplinary team involving at least one liver surgeon, and preferably by a panel of multiple surgeons considering the high rate of disagreement. The implementation of online liver expert panels within oncological networks can help to improve the consistency and quality of care. These panels allow multiple experts to review patients, reducing variability and ensuring that every patient, including those with multiple CRLM, is evaluated for local treatment before and after systemic therapy. This is particularly important given the success of induction therapy in CRLM. Second, consensus guidelines on resection criteria and technical approaches should be further developed to enhance the standardisation of local treatment. Current local treatment evaluation of initially unresectable CRLM remains primarily a technical anatomical decision. Existing clinically available tumour biomarkers fall short in predicting early recurrence after local treatment of CRLM, i.e., selecting patients who will and will not derive benefit from local treatment.^7^ Lastly, most studies in patients with initially unresectable CRLM only report on the percentage of patients in whom local treatment was achieved, and not on the long-term outcome of these patients.^8^ More efforts should be made to identify patients in whom local treatments result in a clinically meaningful benefit. Therefore, further research is needed to explore whether resectability assessments can be supported by other biological resection criteria such as the consensus molecular subtypes and/or circulating tumour DNA. In addition, it is important to examine whether the different technical treatment plans as proposed by the panel surgeons are associated with differences in survival and postoperative morbidity.
